# Health-Risk Behaviour in Deprived Neighbourhoods Compared with Non-Deprived Neighbourhoods: A Systematic Literature Review of Quantitative Observational Studies

**DOI:** 10.1371/journal.pone.0139297

**Published:** 2015-10-27

**Authors:** Maria Holst Algren, Carsten Kronborg Bak, Gabriele Berg-Beckhoff, Pernille Tanggaard Andersen

**Affiliations:** 1 Unit for Health Promotion Research, Department of Public Health, University of Southern Denmark, 6700, Esbjerg, Denmark; 2 Public Health and Epidemiology Group, Department of Health Science and Technology, Aalborg University, 9000, Aalborg, Denmark; Leibniz Institute for Prevention Research and Epidemiology (BIPS), GERMANY

## Abstract

**Background:**

There has been increasing interest in neighbourhoods’ influence on individuals’ health-risk behaviours, such as smoking, alcohol consumption, physical activity and diet. The aim of this review was to systematically review recent studies on health-risk behaviour among adults who live in deprived neighbourhoods compared with those who live in non-deprived neighbourhoods and to summarise what kind of operationalisations of neighbourhood deprivation that were used in the studies.

**Methods:**

PRISMA guidelines for systematic reviews were followed. Systematic searches were performed in PubMed, Embase, Web of Science and Sociological Abstracts using relevant search terms, Boolean operators, and truncation, and reference lists were scanned. Quantitative observational studies that examined health-risk behaviour in deprived neighbourhoods compared with non-deprived neighbourhoods were eligible for inclusion.

**Results:**

The inclusion criteria were met by 22 studies. The available literature showed a positive association between smoking and physical inactivity and living in deprived neighbourhoods compared with non-deprived neighbourhoods. In regard to low fruit and vegetable consumption and alcohol consumption, the results were ambiguous, and no clear differences were found. Numerous different operationalisations of neighbourhood deprivation were used in the studies.

**Conclusion:**

Substantial evidence indicates that future health interventions in deprived neighbourhoods should focus on smoking and physical inactivity. We suggest that alcohol interventions should be population based rather than based on the specific needs of deprived neighbourhoods. More research is needed on fruit and vegetable consumption. In future studies, the lack of a uniform operationalisation of neighbourhood deprivation must be addressed.

## Introduction

From a public health perspective, it is important to reduce social inequalities in health [1]. Previous research has shown that socioeconomic health inequalities have widened in recent decades [2,3]. In the last 20 years, there has been increasing interest in neighbourhoods’ influence on individual health-risk behaviours such as smoking, excessive alcohol consumption, physical inactivity and poor diet [4]. Numerous studies have shown that residents in deprived neighbourhood have higher rates of mortality and morbidity [5–7] than residents of more affluent neighbourhoods, even after taking into account individual-level characteristics such as sex, age, ethnicity and socioeconomic status (SES). Studies have also shown an association between neighbourhood deprivation and health-risk behaviour [6,8]. Most results are from surveys conducted in the USA, the United Kingdom, Canada and the Netherlands [5–8]. Health-risk behaviour is an important factor that increases the risk of morbidity [2] and can explain some of the socioeconomic inequalities in morbidity and mortality [8]. Individuals who engage in four healthy behaviours (fruit and vegetable intake of at least five servings per day, current non-smoker, moderate alcohol intake (1–14 units per week), and physical active) have a life expectancy that is, on average, 14 years longer than that of individuals who do not engage in any of these healthy behaviours [9].

Focusing on health-risk behaviour can be a part of the solution that reduces health inequalities because health behaviour can mediate the effect of SES on the risk of morbidity and mortality [2]. Health-risk behaviour is modifiable through health promotion and intervention programmes; therefore, it is possible to reduce the prevalence and development of these risk behaviours by, for example, developing policies to decrease tobacco smoking, alcohol consumption etc. Through such programmes, it is possible to reduce the gap in life expectancy over the long term [2].

Residents in deprived neighbourhoods are therefore an important target group in efforts to promote healthy behaviour and improve population health in general. “Deprived neighbourhood” is defined here as a geographically bounded area with a high proportion of adults with low SES, as characterised by indicators such as unemployment, low income, low education and low-paying jobs [10]. Knowledge of health-risk behaviour in socially deprived neighbourhoods can contribute to a deeper understanding of the complex interactions between social context, social determinants and health behaviour and to a greater understanding of the development of social inequalities in health behaviour [11]. Through such knowledge, it is possible to develop more targeted health promotion in the process of reducing social inequalities. Previous researchers have investigated how neighbourhood context affects the health of residents by adopting an overall conceptual model in which individual health outcomes are affected by the social and the physical environment of the neighbourhood [4,12–15]. In a review, Diez Roux and Mair have summarised that the social environment of neighbourhoods can affect residents’ health through factors related to safety/violence, social connections/cohesion, local institutions and norms [4]. In addition, they showed that the physical environment can affect health behaviour through environmental exposures, food and recreational resources, the built environment, aesthetic quality/natural spaces, services and quality of housing [4].

The increased interest in neighbourhood effects on individual health is due to, among other factors, multilevel statistical methods, which allow researchers to include both the individual level and the neighbourhood level in one regression model and thereby separate effects related to residents living in the neighbourhood from those related to the neighbourhoods themselves [4,6,16,17].

Previous reviews have primarily focused on examining associations between neighbourhood deprivation and health in general, and these reviews have investigated self-rated health, diseases and health behaviour [6,7,18]. To our knowledge, no systematic review has compared health-risk behaviour among adults in deprived neighbourhoods with that among adults in non-deprived neighbourhoods. By examining the differences in health-risk behaviour in deprived neighbourhoods compared to non-deprived neighbourhoods, it is possible to support future health promotion interventions in deprived neighbourhoods based on which health-risk behaviours warrant the greatest attention.

The aim of this review was to systematically identify and review recent studies on health-risk behaviour among adults who live in deprived neighbourhoods compared with those in non-deprived neighbourhoods. The following research questions were addressed in the present review: 1) What are the differences in health-risk behaviour (no or low consumption of fruits and vegetables, smoking, binge drinking or high-risk alcohol consumption, and physical inactivity) between adults living in deprived neighbourhoods and those living in non-deprived neighbourhoods based on quantitative observational studies and 2) what kind of operationalisations of neighbourhood deprivation were used in the studies?

## Methods

The PRISMA guidelines for systematic reviews were followed as the reporting guidelines for this review [19,20]. There was no protocol for this review.

First, studies were identified by systematically searching electronic databases (PubMed, Embase, Web of Science and Sociological Abstracts) using relevant search terms related to deprived neighbourhoods and health-risk behaviours, Boolean operators, and truncations (see S1 File for a complete list of the search terms used in PubMed). A search strategy was developed and adapted for each database using appropriate subject headings and keywords and was restricted to studies that had been published between 1 January 1996 and 1 July 2014. This period was selected because it was considered to cover the most recent research in health-risk behaviour in deprived neighbourhoods. The search strategies for the Embase, Web of Science, and Sociological Abstracts databases are available upon request (please contact the first author). Second, reference lists in articles for which the full text was assessed were scanned to detect articles that were not found in the database search. The literature search was carried out by the first author (MHA).

### Inclusion criteria

Included studies had to (i) be published in English in peer-reviewed journals; (ii) report data from a primary study that included a sample of a general adult population (16+ years) from deprived neighbourhoods in economically developed Western regions and countries (those from EU-member countries, Andorra, Iceland, Liechtenstein, Monaco, Norway, San Marino, Switzerland, Vatican City, Canada, the USA, Australia and New Zealand); (iii) report how the concept of deprived neighbourhoods was operationalised; (iv) be quantitative observational studies with cross-sectional or longitudinal designs; (v) include health-risk behaviours such as either no or low consumption of fruits and vegetables, smoking, binge drinking or high-risk alcohol consumption, and physical inactivity as outcomes; (vi) compare risk estimates in deprived neighbourhoods with those in non-deprived neighbourhoods; (vii) adjust for at least one confounder besides sex and age; and (viii) be based on data from after 1986 because data prior to 1986 are considered outdated.

### Data extraction and quality assessment

Initially, studies were identified based on titles and abstracts to assess eligibility according to the inclusion criteria. Second, full texts were assessed, and studies were excluded with specific reference to the inclusion criteria. The standardised quality assessment tool for quantitative studies from the Effective Public Health Practice Project (EPHPP) [21] was used to asses risk of bias in all of the reviewed studies; this assessment was performed after the studies were accepted for inclusion in this review (S2 File). This tool is recommended by the Cochrane Collaboration and provides a systematic framework for assessing the quality of studies. Based on the tool, we assessed selection bias, study designs, confounders, data collection methods, and approaches to analyses. The quality assessment helped us to interpret and explain differences in the reported results. The article extraction and quality assessment were performed by one author (MHA). If there was doubt about an article, it was resolved by discussion among all authors.

## Results

**Fig 1:**
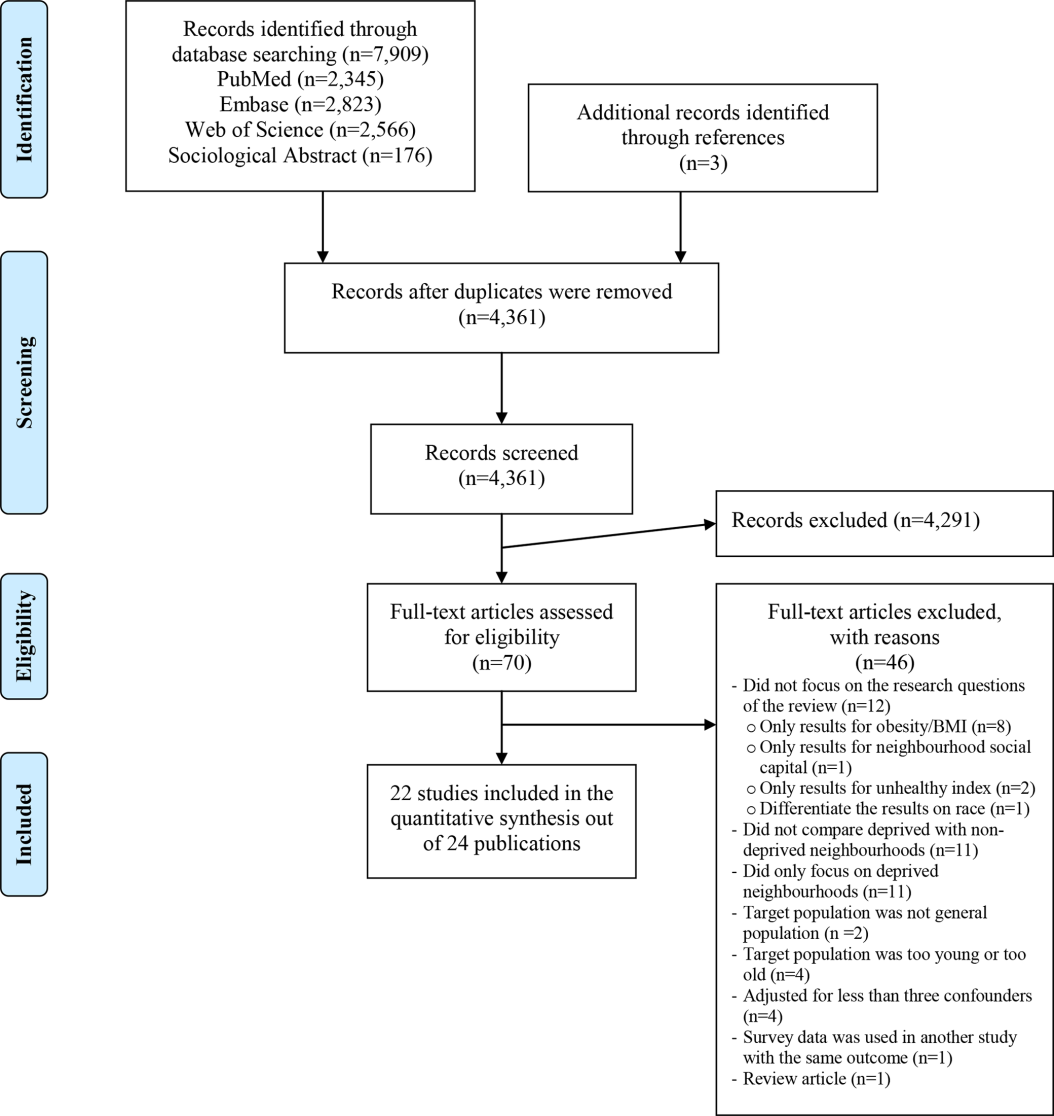
Flow diagram showing the literature search strategy. Flow diagram of the study illustrating identification, screening, eligibility and inclusion processes in the systematic review of health-risk behaviour in deprived neighbourhoods compared with non-deprived neighbourhoods (from the PRISMA statement [19]).

The database searches provided a total of 7,909 citations, and three additional citations were identified through the manual reference search. After duplicates were removed, 4,361 citations remained. Among these, 4,291 citations were excluded because they did not meet the criteria following our review of the titles and abstracts. The full text of the remaining 70 citations was examined, and 46 articles were excluded for different reasons (see Fig 1). In total, 22 studies were included in the systematic review.

The overall quality of the reviewed studies was low. According to the EPHPP criteria, most of the studies had global scores of either “moderate” (n = 10) or “weak” (n = 12). No studies were scored as “strong” (see S2 File).

All included studies had cross-sectional designs (Table 1). Eleven of the 22 studies used structured interviews [8,22–31], and eight studies used self-administered questionnaires [32–39]. Three studies used both methods [40–42]. One study also used clinic biomedical assessment as a supplement [40]. Studies varied widely both in sample size and in the characteristics of the populations studied. The size of the study populations varied from 655 [32] to 58,282 respondents [34]. Nearly all of the studies focused on both men and women, and the sex distribution across most studies was relatively evenly balanced. Most of the results are from studies in the Netherlands (n = 6) [23,24,26,30,32,42], Australia (n = 5) [25,35,38–40], the USA (n = 3) [27,28,33], and the United Kingdom (n = 3) [8,34,37]. Data were collected between 1988 and 2010.

**Table 1:** Characteristics of the reviewed cross-sectional studies.

First author, publication year and country	Study name	Data collection method	Year(s) of data collection (individual level)	Sample and age	Setting	Response rate	Risk bias assessment
Adams et al., 2009, Australia [40]	NWAHS (The North West Adelaide Health Study)	Telephone interview, self-administered questionnaire and clinic biomedical assessment	2000–2002	4,060 adults aged ≥18 years	Northwestern suburbs of Adelaide, South Australia	50%	3
Behanova et al., 2013, Slovak Republic [32]	FP7 EURO-URHIS2 (European Urban Health Indicators Project)	Self-administered questionnaire	2010	655 adults (42.7% men) aged 19–64 years	99 neighbourhoods in cities in the Netherlands	42.6%	3
Cubbin et al., 2006, Sweden [22]	SALAS 1996–2000 (Swedish Annual Level of Living Survey)	Face-to-face interview	1996–2000	18,081 adults (49.2% men) aged 25–64 years	Sweden	80%	3
Diez-Roux et al., 2003, USA [33]	CARDIA (Coronary Artery Disease Risk Development in Young Adults Study)	Self-administered questionnaire	1995/96	3,472 adults aged 28–40 years	USA	79%	2
Dragano et al., 2007, Germany [41]	HNR (Heinz Nixdorf Recall Study)	Computer-assisted personal interview and self-administered questionnaire	2000–2003	4,032 adults (48.5% men) aged 45–69 years	Bochum, Essen, and Mülheim in Germany	56%	3
Fone et al., 2013, UK [34]	WHS (Welsh Health Survey)	Self-administered questionnaire	2003/2004-2007	58,282 adults aged ≥18 years	Wales, UK	74% (2003/2004), 82% (2007)	2
Giskes et al., 2006, Australia [23]	GLOBE sub-sample (Sub-sample of The Dutch GLOBE study)	Face-to-face interview	1991	1,339 adults (50.2% men) aged 25–79 years	85 urban areas in Eindhoven, the Netherlands	80.9%	3
Giskes et al., 2011, Australia [35]	VICLANES (Victorian Lifestyle and Neighbourhood Environment Study)*	Self-administered questionnaires	2003	2,349 adults (43.6% men) aged 18–76 years	Melbourne, Australia	58.7%	3
Kuipers et al., 2013, The Netherlands (1) [42]	POLS 2003–2009 (The Integrated Survey on Living Conditions)	Computer-assisted personal interview questionnaire	2003–2009	26,603 adults aged ≥18 years	963 urban areas in the Netherlands	64–67%	2
Kuipers et al., 2013, The Netherlands (2) [24]	POLS 2004–2009 (The Integrated Survey on Living Conditions)	Computer-assisted personal interview questionnaire and self-administered questionnaire	2004–2009	30,117 adults aged ≥18 years	1722 neighbourhoods across the Netherlands	64–67%	2
Lakshman et al., 2010, UK [8]	EELS^A^ (East of England Lifestyle Survey)	Telephone interview	2008	26,290 adults aged ≥16 years	East of England	11%	3
Migliorini and Siahpush, 2006, Australia [25]	HS^A^ (The Household Survey)	Telephone interview	1990–1997	17,552 (46.9% men) aged ≥18 years	Victoria, Australia	NA	2
Piro et al., 2007, Norway [36]	HUBRO (Oslo Health Study)	Self-administered questionnaire	2000	14,608 adults from five age cohorts: 30, 40, 45 and 60 years	Oslo, Norway	46%	3
Reijneveld, 1998, The Netherlands [26]	NHIS (The Netherlands Health Interview Survey)	Face-to-face interview	1992–1993	5,121 adults aged ≥16 years	An urban setting in Amsterdam, the Netherlands	64.4%	2
Ross, 2000, USA [27]	CCH (Community, Crime and Health)	Telephone interview	1995	2,482 adults (49% men) aged ≥18 years	Illinois, USA	73%	3
Shohaimi et al., 2003, UK [37]	(EPIC-Norfolk) Norfolk component of the European Prospective Investigation into Cancer	Self-administered questionnaire	1993–1997	27,711 adults (45.4% men) aged 39–79 years	A general community in Norfolk, UK	45%	3
Stimpson et al., 2007, USA [28]	NHANES III (The Third National Health and Nutrition Examination Survey)	Face-to-face interview	1988–1994	20,050 adults aged ≥17 years	USA	86%	2
Sundquist et al., 1999, Sweden [29]	SALAS 1988–89 (Swedish Annual Level of Living Survey)	Face-to-face interviews	1988–89	9,240 adults aged 25–74 years	Sweden	80%	3
Thornton et al., 2010, Australia [38]	SESAW (Socioeconomic Status and Activity in Women)	Self-administered questionnaire	2004	1,399 women aged 18–65 years	45 neighbourhoods of varying levels of socioeconomic disadvantage in Melbourne, Australia.	NA	2
Turrell et al., 2010, Australia [39]	HABITAT (How Ares in Brisbane Influence HealTH and AcTivity)	Self-administered questionnaire	2007	11,037 adults aged 40–65 years	200 neighbourhoods in Brisbane, Australia	68.5%	2
van Lenthe et al., 2006, The Netherlands [30]	GLOBE (The Dutch GLOBE study)*	Face-to-face interview	1991	9,062 adults aged 20–75 years	79 neighbourhoods in Eindhoven in the Netherlands	70.1%	2
Wilson et al., 2010, Canada [31]	Hamilton: NA; Glasgow: The West of Scotland Twenty-07 Study: Health in the community (Twenty-07)	Hamilton: Telephone interview; Glasgow: Face-to-face interview	Hamilton: 2000/2001; Glasgow: 2001	Hamilton: 1,203 adults aged ≥ 18 years; Glasgow: 711 adults aged 29–69 years	Hamilton, Canada and Glasgow, Scotland	Hamilton: 60%; Glasgow: 63.4%	3

*Additional articles were published on the same study.

^A^It was not possible to find an existing abbreviation for the study name; thus, we constructed the listed abbreviation.

NA: Not available

The statistical methods of the 22 studies are described in Table 2. Data sources for measuring neighbourhood deprivation consisted of census-defined neighbourhoods (n = 13) [25,27–29,31,33–35,37–41], aggregated self-reported neighbourhood deprivation (n = 2) [23,30] and neighbourhood deprivation based on different public data sources (n = 7) [8,22,24,26,32,36,42]. Most of the studies (n = 18) used population-based respondent selection [8,22–30,32–34,36,37,40–44]; four studies selected their respondents by selecting the neighbourhoods in advance of the surveys [31,35,38,39]. Nineteen studies had multilevel designs [8,22,24–29,32–44], i.e., they included individual-level data nested within the neighbourhood level. Two studies linked individual data with aggregated self-reported neighbourhood characteristics [23,30]. One study used only census data to select the neighbourhoods to survey [31].

**Table 2:** Operationalisations of neighbourhood deprivation and statistical methods in the reviewed cross-sectional studies.

Exposure	Respondent selection	Outcomes^A^	Statistical analysis	Confounders controlled for + age (fully adjusted models)	Study name and reference number
**Census**					
Index of Relative Socio-Economic Disadvantage (IRSD)	Population based	S, AC, PI	Logistic regression	Sex, household income, education, work status/occupation, ethnicity, various health outcomes, lifestyle risk factors	NWAHS [40]
Summary score based on six area variables reflecting the dimensions of wealth/income	Population based	S	Multilevel logistic regression	Sex, income, education, occupation	CARDIA [33]
Unemployment rate and overcrowding	Population based	S, PI	Logistic regression	Sex, education, economic activity, social isolation	HNR [41]
The Welsh Index of Multiple Deprivation	Population based	AC	Multilevel logistic regression	Sex, social class, employment status, education, ethnicity, housing tenure	WHS [34]
Income	Selected neighbourhoods	AC, PI^B^	Multilevel logistic regression	Country of birth, education, occupation, number of people per household, household income	VICLANES [35]
SEIFA Index	Population based	S	Multilevel logistic regression	Education, marital status, employment status, ethnicity	HS [25]
Poverty, education, racial and ethnic composition	Population based	S	Logistic regression	Race, ethnicity, sex, marital status, education, household income, poverty	CCH [27]
Townsend Deprivation Index	Population based	S	Logistic regression	Social class, education, deprivation level	EPIC-Norfolk [37]
Singh Composite Index	Population based	S, AC, PI	Logistic regression	Sex, education, income, employment status, race/ethnicity, marital status, BMI, chronic conditions, sample weight, design effects	NHANES III [28]
Care Need Index (CNI) and Townsend Deprivation Index	Population based	S, PI	Multilevel logistic regression	Sex, education	SALAS 1988–89 [29]
SEIFA Index	Selected neighbourhoods	LFVC	Multilevel logistic regression	Country of birth, marital status, education, occupation, number of dependents, income	SESAW [38]
Index of Relative Socioeconomic Disadvantage (IRSD)	Selected neighbourhoods	PI	Multilevel multinomial logistic regression	Sex, living arrangements, education, occupation, household income	HABITAT [39]
Hamilton: 17 socioeconomic and demographic factors Glasgow: 8 socio- residential factors	Selected neighbourhoods	S, PI	Logistic regression	Sex, occupational social class	NA and Twenty-07 [31]
**Aggregated self-reported**					
Education, occupation, unemployment	Population based	LFC	Multilevel logistic regression	Sex, education, household income	GLOBE sub-sample [23]
Education, occupational level, employment status	Population based	S, PI^B^	Logistic regression	Sex, education, occupation, employment status	GLOBE [30]
**Other publicly available data**					
Unemployment	Population based	LFVC, S, PI	Multilevel logistic regression	Sex, ethnicity, income, education, economic activity	FP7 EURO-URHIS2 [32]
Care Need Index (CNI)	Population based	S, PI	Multilevel logistic regression	Sex, marital status, immigration status, urbanisation, socioeconomic status	SALAS 1996–2000 [22]
18 items including environment problems and SES of residents	Population based	S	Multilevel logistic regression	Sex, ethnicity, household composition, education, income	POLS 2003–09 [42]
18 items including environment problems and SES of residents	Population based	AC	Multilevel logistic regression	Sex, household composition, education, income, population density, social cohesion, percentage of Muslims	POLS 2004–09 [24]
Index of Multiple Deprivation 2007	Population based	LFVC, S, AC	Logistic regression	Sex, ethnicity, employment category, occupational social class	EELS [8]
Composite index of five items: social security benefits, unemployment, disability pension, education, income	Population based	S, PI	Multilevel logistic regression	Sex, marital status, education, employment, income	HUBRO [36]
Income and unemployment	Population based	S	Multilevel logistic regression	Sex, income, occupational status, education	NHIS [26]

*Municipality data, register data, national databases, administrative data sources combined with census data, data from the Oslo City Council, combination of census and self-reported data.

^A^Abbreviations for health risk behaviours: LFVC: Low fruit and vegetable consumption; LFC: Low fruit consumption; LFV: Low vegetable consumption; S: Smoking; AC: Alcohol consumption; PI: Physical inactivity.

^B^Outcomes are published in other articles from the same study.

NA: Not available.

To investigate the association between health-risk behaviour and neighbourhood deprivation, 14 studies used multilevel logistic regression (also called hierarchical modelling) [22–26,29,32–36,38,39,42–44], and the remaining eight studies used binary logistic regression [8,27,28,30,31,37,40,41]. Only hierarchical modelling allows the consideration of data on the individual and contextual levels simultaneously while accounting for the potential dependency of individual observations that share the same characteristics as higher-level variables. Therefore, the results from studies that applied standard regression techniques could be biased. All studies controlled for individual-level confounders; most commonly, these were sex, age, marital status, ethnicity, education, employment status and income.

The operationalisation of neighbourhood deprivation varied widely across studies. Ten studies used different predefined indexes to operationalise neighbourhood deprivation [8,22,25,28,29,34,37–40]. Two studies examined deprived neighbourhoods using SES indicators such as education, occupation, and unemployment, which were aggregates of individual-level variables that had been derived from census and survey data [23,30]. Two studies used a summary score and a composite index of different SES indicators [33,36]. Six studies used a number of different indicators to operationalise neighbourhood deprivation [24,26,27,31,41,42], and two studies used a single indicator [32,35].

Diverse measures were used to assess health-risk behaviour. We broadly categorised the studies according to the following health-risk behaviour outcomes: low fruit and vegetable consumption (n = 4), smoking (n = 16), alcohol consumption (n = 7) and physical inactivity (n = 12). Many studies presented results for multiple outcomes. For more specific definitions of each health-risk behaviour outcome, see Table 3.

**Table 3:** Risk estimates of the reviewed studies for health-risk behaviour in deprived neighbourhoods compared with non-deprived neighbourhoods.

Health-risk behaviour measure	Risk estimate for deprived compared with non-deprived neighbourhoods		Study name and reference number
	OR^A^	95% CI*/p-value	
**Low fruit and vegetable consumption**			
Low fruit and vegetable consumption (<4 servings per day)	1.06	0.70–1.61	FP7 EURO-URHIS2 [32]
Low fruit consumption (<1 portion per day)	0.85^D^	0.58–1.26	GLOBE sub-sample [23]
Low fruit and vegetable consumption (<5 portions on at least 5 day per week)	1.43^F^	1.32–1.56	EELS [8]
Low fruit consumption (<2 or more servings per day)	1.15^F^	0.82–1.61	SESAW [38]
Low vegetable consumption (<2 or more servings per day)	2.33^F^	1.61–3.33	SESAW [38]
**Smoking**			
Current smoker	NA^C^	NA^C^	NWAHS [40]
Daily smoker	1.16	0.56–2.11	FP7 EURO-URHIS2 [32]
Current smoker	3.28	<0.001	SALAS 1996–2000 [22]
Current smoker (at least 5 cigarettes per week)	White: 2.0; Black: 1.1	White: 1.3–3.1; Black: 0.7–1.5	CARDIA [33]
Current smoker	1.60^B^	1.29–1.98	HNR [41]
Current smoker	1.04	0.92–1.18	POLS 2003–09 [42]
Smoker	2.22^F^	1.96–1.44	EELS [8]
Smoker	Women: 1.33; Men: 1.38	Women: 1.13–1.56; Men: 1.17–1.63	HS [25]
Current smoker	1.41	1.21–1.65	HUBRO [36]
Cigarette smoking (≥1 daily)	1.23	1.06–1.43	NHIS [26]
Smoking (smoke 7 or more cigarettes per week)	1.02	<0.05	CCH [27]
Current smoker	Women: 1.86; Men: 1.84	Women: 1.58–2.17; Men: 1.56–2.17	EPIC-Norfolk [37]
Serum cotinine (indicator of smoking)	1.74	1.55–1.96	NHANES III [28]
Current smoker	1.69^E^	1.42–2.01	SALAS 1988–89 [29]
Current smoker	1.24	1.05–1.46	GLOBE [30]
Current smoker	Hamilton: 2.04; Glasgow: 2.40	Hamilton: 1.22–3.41; Glasgow: 1.47–3.91	NA and Twenty-07 [31]
**Alcohol consumption**			
High alcohol intake (women 4≥ and men ≥5–8 drinks per day or occasional excess 9–12 drinks in one day)	NA^C^	NA^C^	NWAHS [40]
Binge drinking (≥6 portions of alcohol at once)	0.95	0.52–1.74	FP7 EURO-URHIS2 [32]
Binge drinking (women >6 units of alcohol at once, men >8 units)	2.21^E^	2.04–2.39	WHS [34]
Consuming alcohol ≥5 days per week	Men: 0.70; Women: 0.77	Men: 0.44–1.12; Women: 0.47–1.28	VICLANES [35]
Medium or high risk of short-term harm (women ≥5, men ≥7 per drinking session)	Men: 1.20; Women: 0.68	Men: 0.80–1.77; Women: 0.46–1.02	VICLANES [35]
Medium or high risk of long-term harm (women ≥15, men ≥29 per week)	Men: 1.11;Women: 0.93	Men: 0.53–2.32; Women: 0.44–1.95	VICLANES [35]
Chronic heavy alcohol use (women ≥14, men ≥21 drinks per week)	0.79	0.61–1.02	POLS 2004–09 [24]
Episodic heavy alcohol use (≥6 drinks a day at least once per week)	0.88	0.67–1.17	POLS 2004–09 [24]
Exceeding recommended limits for alcohol consumption (<22 units per week for men and <15 units per week for women)	0.81^F^	0.76–0.87	EELS [8]
Excessive alcohol consumption (≥5 drinks almost every day)	1.18	1.01–1.38	NHANES III [28]
**Physical inactivity**			
Physical inactivity (<150 min/week moderate activity)	NA^C^	NA^C^	NWAHS [40]
Lack of physical activity (<twice per week)	0.97	0.63–1.50	FP7 EURO-URHIS2 [32]
Physical inactivity (no exercise at all)	3.40	<0.001	SALAS 1996–2000 [22]
Low physical activity (<once per week)	1.25	1.01–1.56	HNR [41]
Low physical activity^G^	0.94^F^	0.89–1.01	EELS [8]
Insufficiently active for health (<150 minutes of activity during the previous week)	1.43^F^	1.11–1.89	VICLANES [43]
No cycling in the last month for 10 minutes or more	1.11^F^	0.79–1.79	VICLANES [43]
No jogging in the last month for 10 minutes or more	1.45^F^	1.06–1.96	VICLANES [43]
No exercise	1.55^F^	1.37–1.75	HUBRO [36]
No physical activity in the past month (such as running, aerobics, yard work, dancing, weightlifting, bicycling, swimming, calisthenics, or any other sport or exercise)	1.52	1.37–1.69	NHANES III [28]
No physical activity	1.61^E^	1.34–1.93	SALAS 1988–89 [29]
Low total activity (MET. minutes/week)	1.78^F^ ^H^	1.34–2.38	HABITAT [39]
Almost never walking, cycling or gardening in leisure time	1.36	1.10–1.69	GLOBE [44]
Almost never participating in sports	1.55	1.33–1.81	GLOBE [44]
0 physically active days	Hamilton: 2.53; Glasgow: 2.40	Hamilton: 1.18–5.43; Glasgow: 1.19–3.41	NA and Twenty-07 [31]

^A^OR, adjusted odds ratio.

^B^The estimate is only presented in regard to neighbourhood unemployment rate.

^C^Only OR differentiated on education.

^D^The estimate is adjusted for sex, age, and education. The estimate adjusted for household income instead of education is similar.

^E^Estimates are calculated based on numbers from the original article.

^F^Estimates from the original article reported the odds of not engaging in the specified risk behaviour. To ensure comparability, we converted the estimates such that the OR would reflect the risk of engaging in the health-risk behaviour.

^G^Not meeting any of the following criteria: ≥3 days of vigorous activity of ≥20 min per day or ≥5 days of moderate-intensity activity or walking ≥30 min per day or ≥5 days of any combination of walking, moderate-intensity or vigorous-intensity activities achieving ≥600 MET-min/week.

^H^We converted the reference group from deprived to non-deprived to ensure comparability.

*CI: Confidence interval.

NA: Not available.

As presented in Table 3, the studies were grouped by type of health-risk behaviour to allow for a more straightforward comparison of the different behaviours. Two out of four studies found a positive association between low consumption of fruit and vegetables and living in deprived neighbourhoods compared with non-deprived neighbourhoods [8,38]. The majority of the studies found a positive association between current smoking and living in deprived neighbourhoods [8,22,25–31,33,36,37,41]. Among the seven studies that examined alcohol consumption, the results were ambiguous. In regard to binge drinking, two studies found a positive association between binge drinking and living in deprived neighbourhoods [28,34]. The East of England Lifestyle Survey found a negative association of exceeding recommended alcohol consumption limits and living in deprived neighbourhoods [8]. There was a clear association between living in deprived neighbourhoods and physical inactivity; three-quarters of the studies found a positive association of physical inactivity in these neighbourhoods [22,28,29,31,36,39,41,43,44].

## Discussion

This review, which compared health-risk behaviour among adults in deprived neighbourhoods and those in non-deprived neighbourhoods, found a clear pattern of increased smoking and physical inactivity in deprived neighbourhoods. These results are in line with the conclusions of previous systematic reviews [6,7,18]. Most of the reviewed studies did not specify any causal mechanisms that linked neighbourhood deprivation to health-risk behaviour. It has been stressed that the social and physical environment of neighbourhoods may be important in understanding how neighbourhoods can contribute to health inequalities [4]. A review by Pickett and Pearl noted that neighbourhoods might affect health-risk behaviour directly (i.e., via the effects of simply living in a deprived neighbourhood) or indirectly through mechanisms such as the availability of and access to healthy foods or recreational facilities, normative attitudes towards health behaviour, and social support [6]. The physical and social features of neighbourhoods may, for instance, affect health behaviour through mechanisms involving the experience of stress and the buffering effects of social support and social relations [4,6,28,45]. It appears likely that stress is associated with an increased health-risk behaviour and that access to social support can reduce these behaviours. Stress may lead persons to engage in coping behaviours related to, e.g., unhealthy diet and smoking, and living in a deprived neighbourhood may itself be a source of stress [6,46,47]. Van Lenthe and Mackenback found that neighbourhood stressors mediated neighbourhood and individual socioeconomic inequalities in smoking [30]. Furthermore, previous systematic reviews on how factors of the built environment affect health have concluded that the built environment can significantly influence individuals’ health [48–50]. To understand how the environment can affect health between neighbourhoods, it is relevant to mention the environmental justice framework. This is a conceptual model that hypothesises that environmental exposures are unequally distributed across social classes and that neighbourhoods or residents with low SES are more vulnerable to environmental exposures [48].

No clear differences between deprived and non-deprived neighbourhoods were found in relation to fruit and vegetable consumption or alcohol consumption. With regard to fruit and vegetable consumption, it should be mentioned that there were only four studies on the subject, which may explain the few significant results. Only one study showed that fruit and vegetable consumption is decreased in deprived neighbourhood. Another study found the same association for vegetable consumption but not for fruit consumption. Furthermore, two cross-sectional studies did not show any association. Therefore, these results should be interpreted with caution. There is a need for more research on fruit and vegetable consumption. In relation to alcohol consumption, we did not find any geographical pattern between countries that might explain the equivocal findings. All studies except for one analysed alcohol consumption adjusted for ethnicity or the proportion of Muslim residents; thus, these factors cannot explain the results. In a systematic review on disadvantaged areas and substance use outcomes, Karriker-Jaffe reported equivocal findings for alcohol consumption [18]. Furthermore, other studies showed that abstaining from alcohol and moderate drinking are more prevalent in deprived versus non-deprived neighbourhoods [8,51]. It is important for future research to consider that alcohol consumption is one of the leading risk factors for mortality and morbidity [52,53]. We highly advise that future interventions combat risky alcohol consumption in all populations. Our findings suggest that the situation in regard to alcohol consumption is not worse in deprived neighbourhoods, which could suggest that interventions can be population-based without considering the specific needs of deprived neighbourhoods. However, these suggestions need to be followed up and tested in future research. Additionally, future research should bear in mind that alcohol consumption is complex in terms of the interaction between age and education level. For example, in Denmark, in the younger age groups, high alcohol consumption is most evident among persons with elementary school as their highest education level; by contrast, in the 65 years or older age group, it is most evident among persons with higher education [54].

Neighbourhoods were operationalised using a variety of techniques. The majority of the studies operationalised neighbourhoods using statistical (e.g., census tracts) or administrative spatial units (e.g., city-defined neighbourhoods). Some studies used multiple neighbourhood socioeconomic characteristics to rank neighbourhood deprivation. The VICLANES [35] and FP7 EURO-URHIS2 [32] used only income and unemployment, respectively, as neighbourhood deprivation measures, and others used indexes. Among the studies using different indicators, the measures that were most often used were income, employment and education. It appears that the indicators used were mainly based on the availability of data rather than on conceptual considerations. Most studies were not explicit about why certain indicators were selected to measure neighbourhood deprivation. Moreover, most studies provided little or no information on the validity and reliability of the measures used.

Neighbourhood deprivation is a frequently used term, but it has no singular definition or operationalisation, as shown in this review. Future research should focus on how to define and operationalise neighbourhood deprivation, which will facilitate systematic review and allow for meta-analysis. However, despite an accepted definition, the problem of how neighbourhood deprivation is operationalised will remain because many researchers must rely on data availability. In the future, researchers should choose neighbourhood deprivation indicators that have been validated. None of the reviewed studies stated whether the measures of neighbourhood deprivation that they used had been validated. A search of the different measures of neighbourhood deprivation used in the reviewed studies did only reveal documentation in regard to the validation of the SEIFA Index [55], which was used in the HS [25] and SESAW [38] studies. In the reviewed studies, data on income, employment and education were frequently used, although there is no true consensus in the literature that these are the best measures of neighbourhood deprivation [56]. Researchers should provide their reasons, both practical and theoretical, for choosing specific measures of neighbourhood deprivation.

Furthermore, the reviewed studies used different labels for neighbourhoods, such as “community”, “area”, and “place”. In general, there is no clear distinction between these terms, and the concepts of neighbourhood and community are not precise [57].

In the current review, we included only studies that at least adjusted for one confounder besides sex and age to control for sociodemographic and socioeconomic differences between respondents living in deprived and non-deprived neighbourhoods. We considered ethnicity and educational level as two of the main potential confounders that could influence the results of this review because these factors have been found to constitute important determinants of health behaviours [58].

We used the EPHPP risk of bias tool to assess the quality of the reviewed studies. Most studies were scored with a global rating of either “moderate” or “weak”, partly because many of the studies (n = 8) had response rates below 60% and all had cross-sectional designs. Accordingly, no causal pathway can be interpreted, and only assumptions about associations are possible. Future studies should, when possible, use cohort designs to capture the long-term effects of neighbourhood deprivation on health-risk behaviour to explicitly examine causal processes over time.

We cannot be certain that all of the reviewed studies included the most deprived neighbourhoods because the data used in most of the studies were based on general health surveys merged with census data, and we know that residents of deprived neighbourhoods are less likely to participate in research [59]. Only four of the reviewed studies [31,35,38,39] selected the deprived neighbourhoods in advance. It is important for future research on deprived neighbourhoods to make a greater effort (e.g., using interpreters in interviews and making multiple contact attempts) to obtain higher resident response rates [60].

A limitation of this review is that it includes only peer-reviewed, English-language articles that could be found in the four selected databases. However, we believe that our search ensured robust data collection because we checked references. Another weakness of this study is that the screening process for selecting and excluding studies was performed by one researcher only due to resource constraints, and this could have potentially reduced the objectivity of study inclusion. In addition, it was not possible for us to conduct a meta-analysis because of the different operationalisations of neighbourhood deprivation and different definitions of health-risk behaviour, which also prevented us from performing a specific check of publication bias such as a funnel plot. However, publication bias cannot be excluded.

The strength of this study is that it gives an update of the research in the field of neighbourhood deprivation and health-risk behaviour from economically developed Western countries in the period between 1996 and 2014. Furthermore, the study emphasises the lack of a definition and operationalisation of neighbourhood deprivation. In addition, we used a very broad search string, which made our searches sensitive and ensured the identification of as many relevant studies as possible.

## Conclusions

Based on the studies that were included in this review, there is consistent evidence that smoking and physical inactivity are more prevalent among adult residents in deprived neighbourhoods than among residents in non-deprived neighbourhoods. No clear differences between deprived and non-deprived neighbourhoods were found in relation to low fruit and vegetable consumption or alcohol consumption, and the results were equivocal. The reviewed studies used different operationalisations of neighbourhood deprivation.

Future health prevention interventions in deprived neighbourhoods should specifically focus on smoking and physical inactivity. We suggest that alcohol interventions should be population-based without considering the specific needs of deprived neighbourhoods.

Future research in this area should address fruit and vegetable consumption. The lack of a uniform definition and operationalisation of neighbourhood deprivation should also be addressed. An understanding of the mechanisms by which neighbourhood deprivation in general affects health-risk behaviour is still lacking. Future research is therefore needed to identify mediators of the association between neighbourhood deprivation and health-risk behaviour. A better theoretical and empirical understanding of these mechanisms or environmental justice will be important for developing and designing more targeted and prioritised health promotion interventions in the process of reducing social inequalities in health. Furthermore, examinations of which groups are most affected by neighbourhood deprivation would be valuable for developing interventions for the most at-risk residents. Additionally, there is a need for more Scandinavian research in this area, as Scandinavia was underrepresented among the reviewed studies.

## Supporting Information

S1 PRISMA Checklist Reported items according to the PRISMA Checklist. (DOC)

S1 File Search string in PubMed. (PDF)

S2 File Quality assessment of the reviewed cross-sectional studies. (PDF)
