# Supplementary material for: Health-Risk Behaviour in Deprived Neighbourhoods Compared with Non-Deprived Neighbourhoods: A Systematic Literature Review of Quantitative Observational Studies
Source: PLoS One. 2015 Oct 27;10(10):e0139297. doi: 10.1371/journal.pone.0139297 (PMC4624433; doi:10.1371/journal.pone.0139297)
Supplement: S1 File — (PDF) [file pone.0139297.s002.pdf]

S1 File. Search string in PubMed

#13 Search **#3 AND #4 AND #12**

#3 Search **((disadvantage*) OR deprived) OR deprivation**

#4 Search **(((Neighbourhood*) OR Neighborhood*) OR Communit*) OR Area***

#12 Search **(((((((((((((((Health behaviour*) OR Health behavior*) OR Diet) OR Fruit) OR Vegetable) OR Smoking) OR "Tobacco use") OR "Alcohol drinking") OR "Alcohol use") OR "Alcohol consumption") OR "Binge drinking") OR "Physical activity") OR "Physical inactivity") OR "Body Mass Index") OR BMI) OR Overweight) OR Obesity**
