# Supplementary material for: Health-Risk Behaviour in Deprived Neighbourhoods Compared with Non-Deprived Neighbourhoods: A Systematic Literature Review of Quantitative Observational Studies
Source: PLoS One. 2015 Oct 27;10(10):e0139297. doi: 10.1371/journal.pone.0139297 (PMC4624433; doi:10.1371/journal.pone.0139297)
Supplement: S2 File — (PDF) [file pone.0139297.s003.pdf]

S2 File. Quality assessment of the reviewed cross-sectional studies*

Author and publication year	Selection bias	Study design	Control for confounding	Data collection	Global rating
Adams et al. 2009	3	3	1	2	3
Behanova et al., 2013	3	3	1	2	3
Cubbin et al., 2006	2	3	1	3	3
Diez-Roux et al., 2003	2	3	2	2	2
Dragano et al. 2007	3	3	1	2	3
Fone et al., 2013	2	3	1	2	2
Giskes et al., 2006	2	3	3	2	3
Giskes et al., 2011	3	3	1	2	3
Kuipers et al., 2013 (1)	2	3	1	2	2
Kuipers et al., 2013 (2)	2	3	1	2	2
Lakshman et al., 2010	3	3	2	1	3
Migliorini and Siahpush, 2006	2	3	2	2	2
Piro et al., 2007	3	3	2	2	3
Reijneveld, 1998	2	3	2	2	2
Ross, 2000	2	3	1	3	3
Shohaimi et al., 2003	3	3	3	2	3
Stimpson et al., 2007	1	3	1	2	2
Sundquist et al., 1999	1	3	3	2	3
Thornton et al., 2010	2	3	2	1	2
Turrell et al., 2010	2	3	2	1	2
van Lenthe et al., 2006	2	3	2	2	2
Wilson et al., 2010	2	3	3	3	3

* The studies could be rated as either Strong (1), Moderate (2) or Weak (3) in regard to each component.
